# Resequencing of 410 Sesame Accessions Identifies SINST1 as the Major Underlying Gene for Lignans Variation

**DOI:** 10.3390/ijms24021055

**Published:** 2023-01-05

**Authors:** Senouwa Segla Koffi Dossou, Shengnan Song, Aili Liu, Donghua Li, Rong Zhou, Muez Berhe, Yanxin Zhang, Chen Sheng, Zhijian Wang, Jun You, Linhai Wang

**Affiliations:** 1 Key Laboratory of Biology and Genetic Improvement of Oil Crops, Ministry of Agriculture and Rural Affairs, Oil Crops Research Institute of the Chinese Academy of Agricultural Sciences, Wuhan 430062, China; 2 Laboratory of Plant Physiology and Biotechnologies, Faculty of Sciences, University of Lomé, Lomé 01BP 1515, Togo

**Keywords:** sesame, genome resequencing, lignan, genomic signatures, NST1, GWAS

## Abstract

Sesame is a promising oilseed crop that produces specific lignans of clinical importance. Hence, a molecular description of the regulatory mechanisms of lignan biosynthesis is essential for crop improvement. Here, we resequence 410 sesame accessions and identify 5.38 and 1.16 million SNPs (single nucleotide polymorphisms) and InDels, respectively. Population genomic analyses reveal that sesame has evolved a geographic pattern categorized into northern (NC), middle (MC), and southern (SC) groups, with potential origin in the southern region and subsequent introduction to the other regions. Selective sweeps analysis uncovers 120 and 75 significant selected genomic regions in MC and NC groups, respectively. By screening these genomic regions, we unveiled 184 common genes positively selected in these subpopulations for exploitation in sesame improvement. Genome-wide association study identifies 17 and 72 SNP loci for sesamin and sesamolin variation, respectively, and 11 candidate causative genes. The major pleiotropic SNP^C/A^ locus for lignans variation is located in the exon of the gene *SiNST1*. Further analyses revealed that this locus was positively selected in higher lignan content sesame accessions, and the “C” allele is favorable for a higher accumulation of lignans. Overexpression of *SiNST1^C^* in sesame hairy roots significantly up-regulated the expression of *SiMYB58, SiMYB209, SiMYB134, SiMYB276*, and most of the monolignol biosynthetic genes. Consequently, the lignans content was significantly increased, and the lignin content was slightly increased. Our findings provide insights into lignans and lignin regulation in sesame and will facilitate molecular breeding of elite varieties and marker-traits association studies.

## 1. Introduction

Sesame (*Sesamum indicum* L., 2n = 26) is a member of the Pedaliaceae family and one of the most important oilseed and industrial crops worldwide. It is primarily cultivated in subtropical and tropical regions in Asia, Africa, Latin America, and Central America [1]. Sesame seeds are promising sources of nutritional components and nutraceuticals for food and pharmacological industries [2,3]. It contains specific lignans, including sesamin, sesamolin, sesaminol, sesamol, etc., that have shown diverse physiological abilities such as anti-cancers, antioxidative, anti-diabetes, anti-inflammatory, anti-hypertensive, anti-proliferative, anti-melanogenesis, anti-depression and memory loss, anti-osteonecrosis, and anti-osteoporosis [4,5,6,7,8,9]. Although it is documented that sesame is the one of the most ancient oilseed crops known to humans, its origins are still a subject of debate, and its progenitor also is not determined [1,10,11,12]. Yu et al. [13] found that the domestication of sesame in China and India might have occurred independently. Other reports indicate that sesame was introduced in China in 2200 BC during the Han dynasty [14]. China is one of the leading countries in terms of sesame production and consumption (www.fao.org/statistics/en/, accessed on 22 November 2021) and has collected and preserved over 7000 accessions in its germplasms [15]. However, sesame’s domestication, population structure, and genetic diversity remain largely unknown.

The release of the sesame reference genome via de novo assembly has allowed genomic studies on sesame biology [12]. The sesame reference genome quality has been improved via the resequencing of 430 recombinant inbred lines [16], which has provided a solid basis for investigating the molecular mechanisms controlling critical quality and agronomic traits of sesame such as high yield, high oil and lignans contents, seed coat color, plant architecture, and melatonin. Significant loci and candidate causative genes underlying these traits have been identified except for lignans variation [15,17,18,19]. The sesame-specific lignans seem to have evolved from defense and accommodation to biotic stresses [20,21,22], suggesting their underlying genes were positively selected during domestication and breeding [13]. Therefore, identifying adaptation-related genes may help understand sesame evolution [23]. The domestication and subsequent breeding of crops generate selective sweeps, eliminating or reducing variation amid the nucleotides adjoining a mutation in DNA [24,25]. Although large-scale genetic variations among diverse accessions of sesame have been reported [15], the detected genomic differentiation between the subpopulations was very low (*F*_ST_ = 0.02), and it was impossible to unravel regions under selective sweeps for exploitation in the crop improvement.

Lignans are a diversified group of phytochemicals that play essential roles in plants’ interactions and adaptations to environmental conditions [26,27]. They are biosynthesized from phenylalanine via the phenylpropanoid pathway and mostly share the same precursors with lignin [26,28,29]. Most naturally occurring lignans are derived from the oxidative coupling of E-coniferyl alcohol moieties [26,28,30]. In sesame, the biosynthetic genes of the major lignans, sesamin and sesamolin have been identified, and their mechanisms of action elucidated [30,31]. Recently, two laccase (*SiLAC1* and *SiLAC39*) and two peroxidase (*SiPOD52* and *SiPOD63*) family genes were identified as the potential pinoresinol synthase genes [32]. However, the regulatory mechanisms of lignan biosynthesis in sesame are largely unknown. The regulation of monolignol biosynthesis is a complex molecular mechanism that involves diverse transcription factors and phytohormones, among which NST1/2 detain pivotal roles [33,34,35]. NST1 and NST2 are master switch regulators of secondary cell wall formation through interaction with diverse family genes, mostly Myb transcription factors [33,34,35]. Therefore, deciphering the complex regulatory network of lignan biosynthesis in sesame is of particular interest for biotechnological applications.

Whole-genome resequencing and population genomic analyses have considerably enhanced the understanding of crops’ genetic variation and domestication history and facilitated marker-traits association studies [36,37,38]. In the present study, we resequenced a worldwide collection of sesame, including 316 accessions from various geographical regions in China and 94 accessions from twenty-eight countries. We investigated the population structure and genetic diversity and revealed key candidate genes under selective sweeps in subpopulations. In addition, the master regulatory gene of lignan and lignin biosynthesis was discovered based on a genome-wide association study (GWAS) and gene expression analyses and validated in sesame hairy roots.

## 2. Results

### 2.1. Genome-Wide Variation and Population Structure

**Figure 1:**
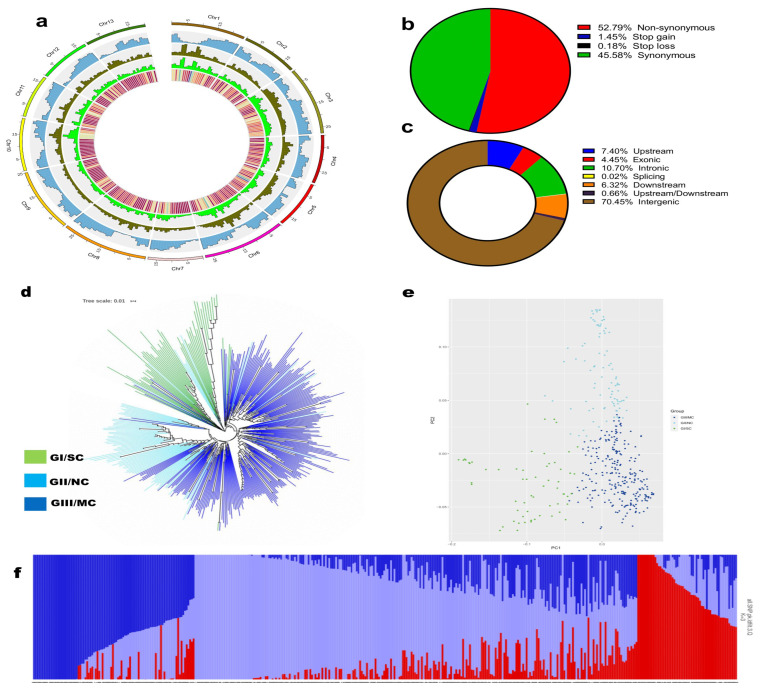
Population structure of sesame accessions in this study. (**a**) Chromosomal distribution of the genomic variants. From the outer to the inner are chromosomes, gene density, SNP density, InDels density, and nucleotide diversity. (**b**,**c**) Classification of the SNPs. (**d**) Phylogenetic tree of all accessions inferred from whole-genome SNPs filtered by linkage disequilibrium (LD) r^2^ < 0.05; The three major groups (Southern cluster, SC; Northern cluster, NC; and middle or central cluster MC) are colored in purple, light blue, and blue, respectively. (**e**) Principal component analysis (PCA) plot for all the sesame accessions; dot colors correspond to the phylogenetic tree grouping. (**f**) Population stratification based on STRUCTURE for K = 3. Each color represents one ancestral population. Each accession is represented by a bar, and the length of each colored segment in the bar represents the proportion contributed by that ancestral population.

We resequenced 410 sesame germplasm samples, including 316 collected from southern, middle, and northern China and 94 collected from 28 countries worldwide representing various geographical regions (Table S1). The population included 329 landraces and 81 modern cultivars. The resequencing generated a total of 3.533 Tb of raw data, with an average of 8.617 Mb per accession (Table S2). The average sequencing depth was 24.93×, and the genome coverage 96.08%. The GC content varied from 36.41 to 40.98% (Table S2). After mapping against the sesame reference genome, we obtained a final set of 5,385,583 single-nucleotide polymorphisms (SNPs) and 1,163,197 insertion-deletion (InDels). The SNPs and InDels are distributed unequally on the 13 chromosomes of the sesame genome (Figure 1a). Figures of 70.45, 10.70, and 4.45% of the SNPs were located in intergenic, intronic, and exonic regions, respectively (Figure 1c). Among the SNPs identified in coding sequences, 45.58% were synonymous SNPs and 52.79% non-synonymous SNPs (Figure 1b). The non-synonymous to synonymous SNPs ratio and the transition/transversion (Ts/Tv) ratio were 1.15 and 1.907, respectively (Table S3).

The SNP-based phylogenetic analysis classified the 410 sesame accessions into three major monophyletic clades which correlated with their geographical distribution (Figure 1d; Table S1). Group I (61 accessions), II (92 accessions), and III (257 accessions) included all the accessions from southern, northern, and central regions, respectively. Therefore, they were labeled SC (southern cluster, located south of 24° N latitude), NC (northern cluster, located north of 32–34° N latitude), and MC (central or middle cluster, located between SC and NC), respectively, to further identify selective sweeps. The landraces and modern cultivars exhibited admixture and were not separated on the phylogenetic tree (Figure 1d; Table S1). The principal component analysis (Figure 1e) and population structure analysis with different K-means levels (Figure 1f and Figure S1) results were identical to the phylogenetic analysis results (Figure 1f and Figure S1).

### 2.2. Genetic Diversity in Sesame Genomes

**Figure 2:**
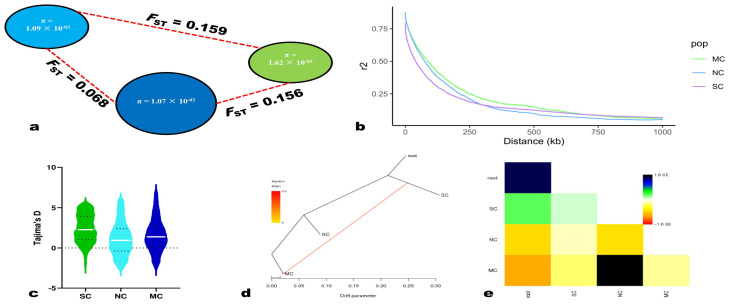
Genetic diversity and gene flow among the subpopulations. (**a**) The diversity (π) and genetic distance (*F*_ST_) across the groups, where green, light blue, and blue colors indicate SC, NC, and MC, respectively; the radius of pie: genetic diversity; and dashed line length: *F*_ST_ value between two groups. SC, NC, and MC represent the subpopulations from the genetic diversity analysis. (**b**) Linkage disequilibrium (LD) differences between subpopulations. (**c**) Distribution of Tajima’s D values. The white line indicates the mean. (**d**) Most frequently found maximum-likelihood trees for gene flow among the subpopulations by Treemix. (**e**) The residual fit values obtained from the maximum likelihood tree (**d**). The plot was constructed using the normalized residual covariances by dividing the residual covariance value between each pair of populations by the standard deviation between all sample pairs. On the right is the color ruler.

To explore genetic divergence among the sesame subpopulations, we performed a pairwise population differentiation (*F*_ST_) across the genome between each group (Figure 2a). The *F*_ST_ value (*F*_ST_ = 0.068) between MC and NC groups is the smallest. The *F*_ST_ value between the SC and MC groups (*F*_ST_ = 0.156) was not too much different from that between the SC and NC groups (*F*_ST_ = 0.159). These results are consistent with the population structure and indicate that the MC and NC group populations have the closest distance and might evolve from the southern subpopulation. We then examined the genetic diversity by computing each group’s nucleotide diversity (π). As we expected, the genetic diversity of the SC subpopulation (π = 1.62 × 10^−3^) is higher than that of the MC group (π = 1.07 × 10^−3^) and the NC group (π = 1.09 × 10^−3^) (Figure 2a). In addition, we explored Tajima’s D values in each group and found a decrease in the average value from southern to northern regions (Figure 2c). We further investigated the linkage disequilibrium (LD) based on r^2^ in the different groups (Figure 2b and Table S4). We found that the distance, when dropped to half of the maximum value, is 89.3 kb for the entire sesame population, which is slightly higher than that reported previously [15]. Finally, we investigated the directions of the gene flow between the subpopulations. The results indicated a “south to middle and north migration model” (Figure 2d,e).

### 2.3. Genomic Selected Signals in MC and NC Groups

**Figure 3:**
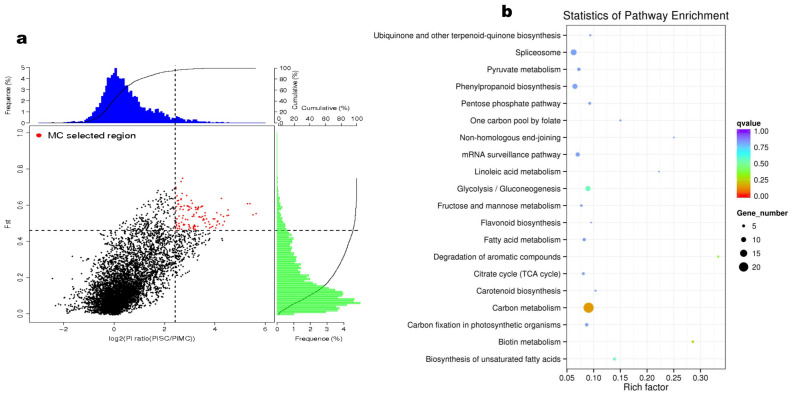
Selective sweeps and functional characterization of positively selected genes in MC group. (**a**) Selection signatures in MC subpopulation. Each red dot indicates a positive selectivegenomic region. (**b**) KEGG (Kyoto Encyclopedia of Genes and Genomes) analysis and enrichment results of genes detected under selective genomic regions in MC subpopulation. The color ruler and *p*-values are shown on the right.

To identify potential candidate genes for exploitation in sesame improvement, we searched for candidate selective genomic regions in MC and NC subpopulations using SC as a background population and adopting the criteria of log_2_(π ratio) > 2.5 and *F*_ST_ > 0.45 (Figure 3a and Figure S3a). We identified 120 and 75 putative selective sweep regions comprising 968 and 571 genes for MC and NC groups, respectively (Tables S5 and S6).

Gene ontology (GO) enrichment analysis for genes selected in the MC subpopulation were mostly related to “structural molecule activity” (GO:0005198), “iron-sulfur cluster binding” (GO:0051536), “metal cluster binding” (GO:0051540), and “protein folding” (GO:0006457) (Figure S2). For the selected genes in NC, the GO terms “transferase activity, transferring phosphorus-containing groups” (GO:0016772), “hydrolase activity, acting on glycosyl bonds” (GO:0016798), “hydrolase activity, hydrolyzing O-glycosyl compounds” (GO:0004553), and “carbohydrate metabolic process” (GO:0005975) were predominant (Figure S3b). KEGG enrichment analysis assigned the MC-selected genes mainly to carbon metabolism, glycolysis/gluconeogenesis, and phenylpropanoid biosynthesis (Figure 3b). Meanwhile, the NC-selected genes were primarily assigned to ribosome, carbon metabolism, glycolysis/gluconeogenesis, phenylpropanoid biosynthesis, and purine and pyruvate metabolism (Figure S3c). We then constructed a Venn diagram among the two sets of selected genes and identified 184 genes (Figure S4). These genes encode diverse enzymes essential for plant development (Table S7).

### 2.4. Genome-Wide Associations for Lignans Variation in Sesame

**Figure 4:**
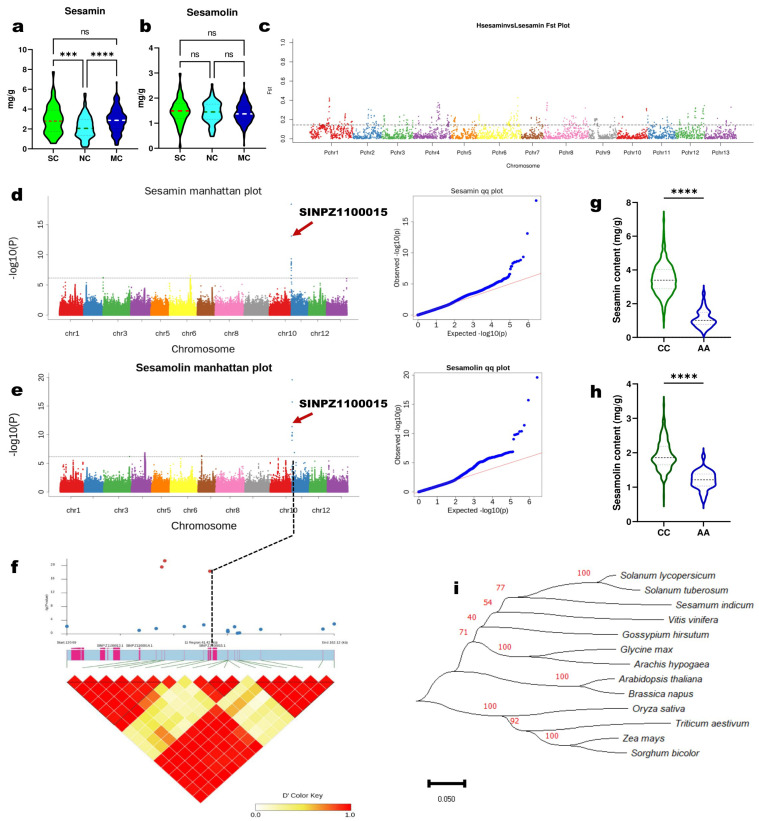
Genome-wide association for sesamin and sesamolin variation. (**a**,**b**) Boxplot of sesamin and sesamolin contents variation among the subpopulations, respectively. MC, NC, and MC indicate the different subpopulations. (**c**) Candidate selective genomic regions in high-sesamin content sesame accessions. *F*_ST_ stands for genetic differentiation. (**d**,**e**) Manhattan and QQ plots for GWAS analysis for sesamin and sesamolin, respectively. The dashed blue line indicates the threshold −log_10_(*p*) = 6. (**f**) LD (linkage disequilibrium) heat map around *SiNST1* (*SINPZ110015*). The dotted line from subfigures e and d to f show the position of *SiNST1* on the sesame genome. (**g**,**h**) Mining of favorable allele for sesamin and sesamolin contents, respectively; accessions with “C” allele, 362 and “A” allele, 34. (**i**) Phylogenetic analysis of *SiNST1* and NST1 from other species. *** *p* < 0.001; **** *p* < 0.0001, *t*-test.

Sesame contains two major and clinically important lignans, sesamin and sesamolin. To dissect the genetic basis of lignans variation in sesame, we cultivated all the 410 accessions in six different environments in Guangxi (GX, 2014), Wuhan (WH, 2014 and 2018), Hunan (HN, 2014), Shandong (SD, 2018), and Zhumadian (ZM, 2019). Seed samples from each location were analyzed by HPLC (high-performance liquid chromatography). The summary of the phenotypic data is presented in Table S8. In general, the sesamin and sesamolin contents varied from 0.06 to 10.65 mg/g and 0.05 to 4.77 mg/g, respectively. The coefficient of variation of sesamin ranged from 33.92 to 45.18%, while that of sesamolin ranged from 21.41 to 28.88%. The frequency distribution of the two traits and their correlations across the six environments are shown in Figure S5. As sesamin and sesamolin contents showed considerable variation, we fitted their values in the six environments to obtain a BLUP (best linear unbiased predictor) value, using a linear mixed model in the lme4 R package. To examine the influence of genetic variation on sesamin and sesamolin contents, we compared their respective values in the subpopulations (Figure 4a,b). We found no significant difference in the sesamolin content of the three groups (Figure 4b). However, the sesamin content in MC and SC was significantly (*p* < 0.001) higher than in NC (Figure 4a). We further investigated the population differentiation (*F*_ST_) between high-sesamin (HS, value > 6 mg/g) and low-sesamin (LS, value < 1 mg/g) accessions. The *F*_ST_ analysis revealed significant divergent genomic regions comprising 2119 genes (Figure 4c; Tables S9 and S10). These genes were mainly related to metabolic processes, especially oxidoreductase activities and cellular processes (Table S10 and Figure S6). We searched for candidate selective regions for HS and LS content. We identified 41 and 65 putative selective genomic regions harboring 363 and 343 genes for HS and LS content, respectively (Tables S11–S14). KEGG annotation and enrichment analysis assigned the HS accessions selective genes mainly to plant-pathogen interaction, peroxisome, pentose and glucuronate interconversions, mRNA surveillance pathway, glycerolipid metabolism, and carbon metabolism (Figure S7a). While the LS accessions selective genes were principally assigned to metabolic pathways and biosynthesis of secondary metabolites (Figure S7b).

We performed a GWAS on the two traits via the MLM (mixed linear model) implemented in the EMMAX (Efficient Mixed-Model Association eXpedited) software. The results showed that the model was adequate for analyzing these important traits (Figure 4d,e). In total, 17 and 72 significant SNP loci (threshold −log(*p*) value > 6) were identified for sesamin and sesamolin, respectively (Table S15). Interestingly, most of the loci locate at our previously detected QTL positions of the two lignans [39]. The major association signal (Chr11-142842) was pleiotropic for the two traits (Figure 4d,e) and is located in the exon of the gene *SINPZ1100015* (*SiNST1/SIN_1005755*), which encodes an NAC domain-containing protein 43. It is homologous to the *Arabidopsis* NST1, which is a master regulator of secondary cell wall deposition through the control of monolignol biosynthesis [33,34,35]. *SiNST1* has shown significant associations with variations in oil, seed coat thickness, and phytosterol contents in sesame [15,40]. In addition, we screened for candidate genes within the LD windows of the other associated loci. By combining gene function annotation and expression patterns in two contrasting sesamin content varieties, we selected ten additional candidate causative genes for lignans variation in sesame (Figure S8 and Table S16).

Interestingly, the locus of *SiNST1* exhibited significant divergence between HS and LS sesame accessions (Figure 4c and Table S10). The results of the regional association (LD) analysis of *SiNST1* ± 10 kb corroborate with the GWAS analysis (Figure 4f). *SiNST1* is primarily expressed in sesame seeds and capsules (Figure S9A). The non-synonymous SNP locus (Chr11-142842) consists of “C” allele change into “A” conducting amino acid change from Thr (threonine) to Lys (lysine). Superior allele analysis showed that the common allele “C” significantly increased (*p* < 0.0001) the sesamin and sesamolin contents than the variant allele “A” (Figure 4g,h). qRT-PCR analysis also showed that the “C” allele is favorable for lignans biosynthesis during seed development (Figure S9B). In sesame accessions with the “C” allele, *SiNST1* is highly expressed at the key stages (10 to 20 DPA) of sesamin and sesamolin biosynthesis [12,30,41]. While in sesame accessions with the “A” allele, *SiNST1* is mostly expressed at the early stages of seed development. Further, we searched for the coding sequence (CDS) of homologs of *SiNST1* in other crops via NCBI. The CDS of *SiNST1* consists of 376 amino acids (aa). Phylogenetic analysis clearly separated NST1 from monocots and dicots. On the phylogenetic tree, *SiNST1* clustered closely to NST1 in *Solanum tuberosum* and *S. lycopersicum* (Figure 4i).

### 2.5. SiNST1 Is a Master Regulator of Lignans and Lignin Biosynthesis in Sesame

**Figure 5:**
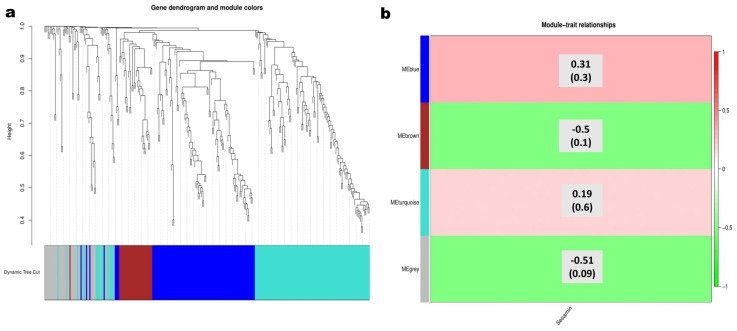
WGCNA results of *SiNST1*, *SiMYBs*, and predicted CYP81Q1-related genes in developing sesame seeds. (**a**) Classification of the analyzed genes in four modules represented by the blue, brown, grey, and turquoise colors. The genes with higher weight values have the most extended height on the dendrogram. (**b**) Heatmap of the correlation of WGCNA modules with sesamin content. The ordinate and abscissa indicate modules and the trait (sesamin), respectively. The green or red colors indicate the correlation coefficients (the top number in each cell). On the right is the color ruler. The bottom numbers (numbers in brackets) indicate the *p*-values.

Since NST1 is reported as a master regulator of lignans and lignin biosynthesis in plants by interacting with hormonal signals and MYB transcription factors, we explored the cis-acting elements in the promoter region of *SiNST1* and *PSS* (piperitol/sesamin synthase). The results showed that *SiNST1* contains mainly MYB binding, light responsiveness, and hormone-related elements (Table S17). *PSS* primarily contains MYB recognition elements and MeJA (methyl jasmonate) responsive elements (Table S18). We further performed a WGCNA (weighted gene correlation analysis) using the expression values (FPKM) of *SiNST1*, the 287 sesame MYB genes (*SiMYBs*) [42], and the 25 predicted CYP81Q1 genes [12], including *PSS*. As shown in Figure 5a, these genes were classified into four subgroups: blue; brown; grey; and turquoise modules. The blue module exhibited the highest positive correlation (r = 0.31) with sesamin and included *SiNST1* (Figure 5b). *SiNST1* appeared as the hub gene in this module and showed co-expression relationships with 85 *SiMYBs* and four CYP81Q1-related genes (Table S19). We found no direct interaction between *SiNST1* and *PSS*. *PSS* was classified into the turquoise module, which also exhibited a positive correlation (r = 0.19) with sesamin content (Figure 5b). The top ten genes in this module included seven MYBs and three CYP81Q1 genes (Table S20).

**Figure 6:**
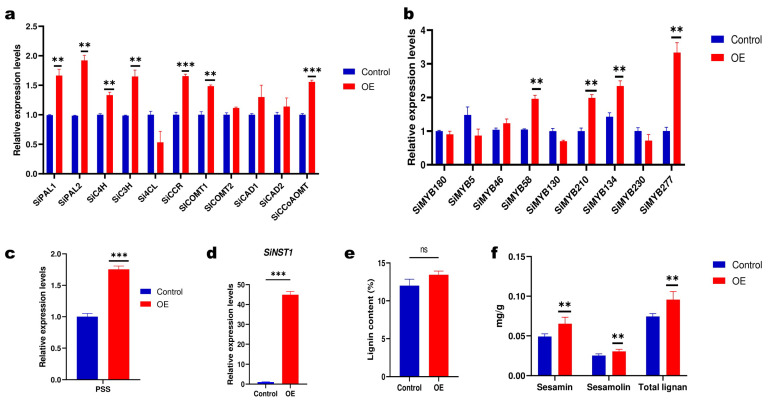
Functional validation of *SiNST1* in sesame hairy roots. (**a**) Expression analysis of monolignol biosynthetic genes. (**b**) Co-expressed *SiMYBs* with *SiNST1* predicted to involve in lignin biosynthesis. (**c**) piperitol/sesamin synthase, *PSS.* (**d**) *SiNST1*. (**e**) Lignin content. (**f**) Lignans content in transgenic sesame hairy roots. *** *p* < 0.001; ** *p* < 0.01; *t*-test.

To validate the potential function of *SiNST1*, we overexpressed *SiNST1^C^* in sesame hairy roots (Figure S10). The *SiNST1^C^*-overexpressing (OE) sesame hairy roots exhibited a significant increase (*p* < 0.01) in the content of sesamin and sesamolin (Figure 6e). As lignans and lignin share the same biosynthetic pathway, we also evaluated the lignin content in the sesame hairy roots. The results indicated a slight increase in the lignin content of OE compared to the control, but the difference was not significant (Figure 6d). To acquire insights into the regulatory mechanism of *SiNST1*, we investigated via qRT-PCR the expression levels of sesame monolignols biosynthetic genes, co-expressed *SiMYBs* predicted to involve lignin or phenylpropanoid biosynthesis, and *PSS*. The results showed that the *SiNST1* overexpression significantly influenced the expression of monolignol biosynthetic genes (Figure 6a). The expression levels of *SiPAL* (phenylalanine ammonia-lyase), *SiC4H* (cinnamate 4-hydroxylase), *SiC3H* (coumarate 3-hydroxylase), *SiCCR* (cinnamoyl-CoA reductase), *SiCOMT* (caffeic acid 3-O-methyltransferase), and *SiCCaAOMT* (caffeoyl-CoA O-methyltransferase), were significantly increased. Among the investigated MYB genes, *SiMYB58*, *SiMYB209*, *SiMYB134*, and *SiMYB276* exhibited a significant increase in their expression levels in OE (Figure 6b). The expression level of *PSS* in OE was also significantly increased (Figure 6c).

## 3. Discussion

We provide comprehensive large-scale genome-wide polymorphism data in sesame, one of the most ancient oilseed crops with high economic, cultural, nutritional, and therapeutical values worldwide, especially in East Asia. The 410 sesame accessions resequencing data provide insights into the population structure and high throughput markers (5.38 and 1.16 million SNPs and InDels, respectively) for genotype selection and association studies to unveil key regulatory genes and dissect complex molecular mechanisms underlying agronomical and quality traits. The non-synonymous to synonymous SNPs ratio was 1.15, which is smaller than in soybean (*Glycine max* L., 1.61) [38], castor (*Ricinus communis* L., 1.39) [43], Tartary buckwheat (*Fagopyrum tataricum* L., 1.69) [44], and chickpea (*Cicer arietinum* L., 1.20) [45]. The transition/transversion (Ts/Tv) ratio of 1.907 for sesame is smaller than that for Tartary buckwheat (2.175) [44] but higher than that for maize (*Zea mays* L., 1.02) [46], tomato (*Solanum lycopersicum* L., 1.75) [47], and black gram (*Vigna mungo* L., 1.58) [48].

The domestication and evolution histories of sesame are unclear and are expected to be explored after the whole-genome sequencing of wild sesame plants [1]. Here, our investigations (phylogenetic and PCA analyses) show that sesame accessions were geographically different and exhibited a pattern of SC, MC, and NC. The landraces and modern cultivars exhibited admixture, which is consistent with the previous report by Wei et al. [15]. The population differentiation (*F*_ST_), genetic diversity, and gene flow analyses indicated that the SC subpopulation is the oldest sesame population, and the MC and NC clades emanated from the SC group. The genetic distance (*F*_ST_) between NC and MC groups was very low compared to their respective genetic distance with the SC group. The MC and NC subpopulations have relatively low genetic diversity (π) compared with the SC group. These results suggest that the SC subpopulation might be relatively close to wild sesame species. Moreover, the variation in genetic diversity suggests different intensities of genes selection during geographical differentiation. We identified 184 common genes under significant selective genomic regions in MC and NC groups. These genes encode diverse enzymes essential for plant development and may represent target genes for improving sesame quality traits and environmental adaptation. However, these potential environmental adaptation-related genome markers and candidate genes need to be confirmed via a Genome-Environment Association (GEA) and subsequently validated through functional genomics studies [49,50]. Otherwise, as the studied population contained majority accessions from China, our findings could imply that sesame introduction and domestication in China commenced in a locality in southern China and later extended to middle and northern China. Supportively, similar domestication events have been reported in soybean and Chinese castor [38,43]. This extensive genomic variation and substantiation of genetic differentiation amid regional subpopulations will favorize structured association mapping and the design of breeding programs that will seize the entire diversity in sesame [51,52,53].

Sesame lignans have become compounds of considerable scientific research interest due to their biological properties and huge importance [54,55,56,57]. Accordingly, the main current objective in sesame breeding is to create elite varieties containing high oil and lignans. The genetic variants governing natural variation in oil content and composition have been explored [15,58,59]. Selective sweeps analysis identified 363 positive selected genes in high-sesamin content sesame accessions. Functional analysis assigned these genes mostly in plant-pathogen interaction, peroxisome, pentose and glucuronate interconversions, mRNA surveillance pathway, glycerolipid metabolism, and carbon metabolism. These findings indicate that sesamin and sesamolin might be primarily involved in sesame biotic stress tolerance mechanisms. We found that the sesamin and sesamolin contents varied considerably among the six environments, and the content of sesamin significantly decreased from SC to NC. These results show genetic selection, domestication, and environmental conditions have significant impacts on sesame agronomic traits. We mainly focused on association analysis for sesamin and sesamolin variation and identified significantly associated loci and major candidate effect genes. The genetic polymorphism within the exonic region of the gene *SiNST1* may contribute substantially to lignan variation in sesame. Favorable allele mining provides important resources for marker-assisted selection. *SiNST1* encodes for an NAC domain-containing protein 43 and was positively selected in the high-sesamin content sesame accessions. Its homolog *AtNST1* in *Arabidopsis* is a master switches transcriptional regulator of SCW (secondary cell wall) formation by interacting with some MYB genes and hormonal signals [33,34,35]. Phylogenetic analysis clearly separated NST1 from monocots and dicots, indicating the gene might play different functions in the two categories of Angiosperm. On the phylogenetic tree, *SiNST1* clustered within dicots closely to NST1 in *Solanum tuberosum* and *S. lycopersicum*, indicating it might fulfill similar functions in sesame. *Cis*-acting elements analysis revealed that the promoter region of *SiNST1* mainly contains MYB binding, light responsiveness, and hormone-recognizing elements. These results suggest that *SiNST1* might regulate sesamin and sesamolin biosynthesis through interplays with MYB genes and phytohormones as per its homologous. Consistency, WGCNA revealed that *SiNST1* might interact with diverse *SiMYBs* in developing sesame seeds, confirming it might regulate diverse developmental processes in sesame. However, no direct interaction between *SiNST1* and *PSS* was noticed. *SiNST1* is also significantly associated with seed coat thickness, phytosterol content, and oil content in sesame [15,40]. The non-synonymous SNP locus in *SiNST1* consists of “C” allele change into “A” resulting in an amino acid change from Thr (threonine) to Lys (lysine). Sesame accession with the “C” allele accumulated significantly higher sesamin and sesamolin compared to accessions with the “A” allele. Supportively, gene expression analysis showed that *SiNST1^C^* is mainly expressed during lignan biosynthesis stages of 10 to 20 DPA, while *SiNST1^A^* is primarily expressed at seed coat development stages (early stages) [12,15,30]. These findings show that *SiNST1^C^* is a potential target for improving nutrient content in sesame, while *SiNST1^A^* may favorize seed coat thickness [15]. Together, our findings suggest that the thicker the sesame seed, the less oil and lignan may it contain. However, it should be noted that only 35 accessions with the “A” allele were contained in the studied population. Therefore, a future study is required to analyze the impact of nucleotide variability of *SiNST1* on sesame seed quality traits.

Since an efficient genetic transformation method for the sesame plant is not yet established, we overexpressed *SiNST1^C^* in sesame hairy roots to investigate its regulatory mechanisms. The overexpression of *SiNST1^C^* significantly induced the expression of *SiMYB58*, *SiMYB209*, *SiMYB134*, *SiMYB276*, and most monolignol biosynthetic genes. Consequently, the *SiNST1^C^* -overexpressing sesame hairy roots exhibited an increase in lignan and lignin contents. However, the increase in the lignin content was not significant. These findings indicate that *SiNST1* plays a master switches function in regulating SCW formation and lignan biosynthesis in sesame by interacting with these four *SiMYBs*. Moreover, we identified ten other candidate genes for sesamin and sesamolin variation and seven *SiMYBs* that showed significant co-expression correlation with PSS in the turquoise module. These seven *SiMYBs* might be the key regulator of *PSS* expression. Therefore, further functional characterization of *SiNST1* and these genes in developing sesame seeds via subsequent knockout and overexpression is required when a genetic transformation protocol will be established to dissect this complex molecular regulatory network for biotechnological applications. Lignans and lignin are principally biosynthesized from the oxidative coupling of E-coniferyl alcohol, a critical reaction catalyzed by dirigent proteins (DIRs) [26,28,60,61]. Therefore, genome-wide characterization of the DIR gene family and identification of *SiDIRs* that may promote lignan accumulation in sesame are required for targeted balancing of the oxidation coupling in favor of lignan biosynthesis. In addition, identifying key quality-related candidate genes in wild sesame accessions will offer supplementary resources for sesame improvement. For instance, Wang et al. have found that the gene CYP92B14 promotes high sesamolin accumulation in wild allotetraploid sesame (*S. schinzianum*) [62]. This gene has to be functionally characterized for exploitation in genomic-assisted breeding of high sesamin and sesamolin content sesame varieties.

## 4. Materials and Methods

### 4.1. Plant Materials and Genome Sequencing

The 410 sesame accessions (329 landraces and 81 modern cultivars) were given by the National Mid-term Gene Bank, Oil crops Research Institute, Chinese Academy of Agricultural Sciences (OCRI, CAAS), Wuhan, China. The population is composed of 316 accessions from diverse geographical regions in China and 94 accessions from twenty-eight other countries (Table S1). Related to the type of variety, there were 329 and 81 landraces (local varieties) and modern cultivars (improved via breeding), respectively. Each accession sample was maintained via cultivation and self-pollination for over three generations prior to sequencing and phenotypes evaluation. A representative plant of each accession was chosen for the genome sequencing. Genomic DNA was extracted from leaves at the seedling stage via the CTAB method [63] and following the manufacturer’s instructions. The sequencing library was constructed from high-quality DNA with an insert size of ~350 bp. The paired-end sequencing of each accession library was carried out on the Illumina HiSeqTM/MiSeqTM platform (Novogene Co., Ltd., Beijing, China).

### 4.2. Sequence Alignment, Variants Detection, and SNP Annotation

All the paired-end reads were mapped onto the sesame reference genome (version 2.0) [16] using the BWA (Burrows-Wheeler Aligner) software [64] with default parameters in order to locate the position of each read on the sesame genome. Next, we filtered out the low-quality (MQ < 20) reads with the SAMtools (v1.1) [65] program. We sorted and discarded PCR duplicates to construct a bam file index using Picard Tools (http://broadinstitute.github.io/picard/; v1.118, accessed on 2 March 2021). Finally, we called the genome variants (SNPs and InDels) with GATK (Genome Analysis Toolkit) [66], as described by Fan et al. [43]. To evaluate the quality of the identified SNPs, we randomly selected fifteen sesame accessions and repeated the whole-genome sequencing. The consistency of the results was approximately 99.8%.

The SNP annotation was conducted according to the sesame reference genome with ANNOVAR [67]. First, the SNPs were classified into upstream or downstream regions, intronic, exonic, intergenic, and splicing sites. Further, SNPs located in the exonic areas were grouped into synonymous and non-synonymous SNPs.

### 4.3. Population Genetic Analyses

The effective, high-quality SNPs were sorted using the criteria of Min depth ≥ 8x, MAF (Minor allele frequency) ≥0.05, and missing rate ≤0.2. Phylogenetic, PCA, structure, and LD analyses allow the examination of the evolutionary history or relationship between individuals of a population and characterize the population stratification and genetic variation. We constructed the approximate maximum-likelihood tree using the general time-reversible model in the TreeBeST (http://treesoft.sourceforge.net/treebest.shtml, accessed on 12 June 2021) software [68]. The population structure analysis was conducted in the Admixture program [69] with various K values. PCA (principal component analysis) was performed in the GCTA software [70]. The dataset used to conduct the PCA was screened for quality using the criteria of scaffold length >100 kb and (QUAL in the VCF file) <2000. Linkage disequilibrium (LD) decay analysis was performed in the PopLDdecay software [71].

To gain insight into the genetic diversity of the sesame population, we computed the genetic distance (*F*_ST_) and nucleotide diversity (π) using VCFtools [72] with a 10 kb nonoverlapping window slid across each scaffold. The *F*_ST_ between clades was evaluated using the average value of all 10 kb sliding windows. The selective sweeps of geographical differentiation of SC subpopulation against other groups were revealed by considering both the *F*_ST_ value (*F*_ST_ > 0.45) and the π ratio (log_2_(π ratio) > 2.5) in each sliding window, with a 5% cutoff [43]. The intergroup gene flow analysis was performed in Treemix [73], using a composite-likelihood approach. We defined the root via the root parameter and tested for many migration events to select the most frequent ones with significant (*p* < 0.05) gene flow.

### 4.4. Gene Ontology (GO) and KEGG Analyses

The gene annotation was conducted with PFAM terms using InterProScan 5 (http://www.ebi.ac.uk/Tools/pfa/iprscan5/, accessed on 19 September 2020) as per Hazzouri et al. [52]. Functional annotation and categorization of genes were achieved through gene ontology (GO) and KEGG analyses. The GO terms and the KEGG (Kyoto Encyclopedia of Genes and Genomes) analyses were carried out using AutoFact [74] and the Automated Annotation Server (KAAS; http://www.genome.jp/tools/kaas/, accessed on 9 August 2021), respectively. The ggplot2 package in R was used to sort the significant terms (*p*-value < 0.05) [75].

### 4.5. Phenotyping

The two primary lignans in sesame, sesamin and sesamolin, were investigated in six environmental conditions located in China, including Guangxi (GX, 2014), Wuhan (WH, 2014 and 2018), Hunan (HN, 2014), Shandong (SD, 2018), and Zhumadian (ZM, 2019). Wuhan belongs to the Yangtze river valley (southern China) with a warm and temperate climate. Zhumadian belongs to the Huang-Huai river valley (northern China) with a humid subtropical climate. Hunan and Guangxi are located at southern China with a subtropical humid monsoon and tropical monsoon climates, respectively. Shandong has a temperate monsoon climate. HPLC analysis of the sesamin and sesamolin contents in seed samples was carried out as previously reported [39,76]. The phenotypic data were analyzed with GraphPad.prism V. 9.0.0121 (GraphPad Software Inc., La Jolla, CA, USA).

### 4.6. GWAS Analysis and Candidate Genes Identification

The association analysis was carried out with the EMMAX (Efficient Mixed-Model Association eXpedited) program via the mixed linear model (MLM) [77], in order to uncover genetic markers that drive lignan variation in sesame. The significant association threshold was set at −log_10_*p* > 6. The Manhattan and QQ plots were constructed in R using the package “qq-man” [78]. The candidate genes in LD windows (±89 Kb) were selected through the integration of gene function annotation, non-synonymous SNPs, and gene expression analysis. Finally, we performed pairwise LD correlation analysis to confirm the major significant associated locus. The pairwise LD correlations analysis of the major candidate causative gene was carried out with LDBLockShow software [79].

### 4.7. Cis-Acting Elements and Phylogeny Analyses

*Cis*-acting elements prediction in the 2000 bp upstream promoter region of the targeted genes was achieved with PLANTCARE (http://bioinformatics.psb.ugent.be/webtools/plantcare/html/, accessed on 21 November 2021), in order to predict their respective potential functions. The protein sequence of *SiNST1* homolog in *Glycine max* (XM_003548125), *Arabidopsis thaliana* (NM_130243), *Brassica napus* (XM_013895547), *Triticum aestivum* (XM_044585881), *Oryza sativa* (XM_015793488), *Sorghum bicolor* (XM_002436372), *Zea mays* (XM_008650556), *Arachis hypogaea* (XM_025776021), *Gossypium hirsutum* (XM_016853342), *Vitis vinifera* (XM_002279509), *Solanum lycopersicum* (XM_004248375), and *S. tuberosum* (XM_015310959) were downloaded by blast from NCBI. These species were selected to represent monocots and dicots and based on previous studies [33,34,35]. The alignment and the phylogenetic analysis were performed with the MEGA X software [80].

### 4.8. WGCNA Analysis

Weighted gene co-expression network analysis (WGCNA) is a system biology method widely used to reveal correlation patterns among genes and identify candidate genes for specific agronomic traits. The WGCNA was conducted using the expression values (FPKM) of *SiMYBs* [42], predicted CYP81Q1 genes [12], and *SiNST1*. The expression values were extracted from available RNA-seq data of two sesame accessions (Zhongzhi16 and ZZM2748) with contrasting lignans content. The co-expression analysis was performed with the WGCNA package in R (version 4.0.2) [81].

### 4.9. Hairy Roots Transformation and Culture

The coding sequence of *SiNST1* was cloned (primers: F-AGAAGAGGGCTGGGTGGTT and R-GGATGACGAGTTGGGGCTAT) from Zhongfengzhi1 (harboring the favorable allele “C” at the locus Chr11-142842) and inserted into the pCAMBIA 1301S vector. The recombinant vector pCAMBIA 1301S-*SiNST1* was transformed to *Escherichia coli* DH5α (Weidi Biotechnology, Shanghai, China). After confirming that the transformation sequence was correct, a suitable amount of plasmid DNA was extracted from the previously transformed *E. coli* and transferred to *Agrobacterium rhizogenes* K599 for subsequent generating transgenic sesame hairy roots following previously described methods [82,83] with some modifications. Sesame sterile seedlings at two weeks old were wounded and used for the *Agrobacterium* infection for 10 min. Next, co-culture in the dark on MS solid medium for 48 h at 25 °C, followed by explants washing by MS liquid medium (containing 300 mg/mL cefotaxime) and sterile water. Then the explants were cultured on MS solid medium containing 200 mg/L timentin in a growth chamber to induce the hairy roots. The induced single sesame hairy root lines were discarded from explants after two weeks and grown on MS solid medium containing 200 mg/L timentin for detoxification. The positively transgenic lines were screened via PCR and grown on MS solid medium for two weeks under a 16/8 h light/dark photoperiod at 28 °C. Hairy roots were harvested, washed, and dried for sesamin, sesamolin, and lignin evaluation.

### 4.10. Lignans and Lignin Contents Analyses in the Sesame Hairy Roots

The extraction method of sesamin and sesamolin from the hairy roots was different. First, 0.3 g of crushed hairy roots was extracted with 5 mL absolute ethanol by shaking for 2 h. After centrifugation at 5000× *g* for 5 min, the supernatant was collected, and the ethanol was evaporated completely at 60 °C. Then, the residue was dissolved in 1 mL ethanol 80% by vortexing for 2 to 3 min. Finally, the extract was filtered and subjected to HPLC analysis. The lignin (acid-soluble) content was evaluated as per Wu et al. via the Klason method [84].

### 4.11. qRT-PCR Analysis

Total RNA from the hairy root samples was extracted with the EASYspin Plus plant RNA Kit (Aidlab, Beijing, China) and, thereafter, reverse transcribed using the HIScript II first-strand cDNA synthesis Kit (Vazyme Biotechnology, Nanjing, China) following the manufacturer’s instructions. The qRT-PCR was carried out with ChamQ™ SYBR1 qPCR Master Mix (Vazyme Biotech, Nanjing, China) on LightCycler480 (Roche, Switzerland) real-time PCR system. The histone H3.3 gene (*SINPZ1301428*) was used as an internal control to normalize the transcript levels [85]. Each gene was analyzed using three biological replicates under the same conditions. The expression levels of the genes were analyzed using the 2^−ΔΔCT^ method [86]. The primers were designed with Primer3 (http://bioinfo.ut.ee/primer3-0.4.0/, accessed on 18 February 2022) and are listed in Additional file 1: Table S21.

### 4.12. Statistical Analysis

GraphPad Prism v9.0.0121 (GraphPad 159 Software Inc., La Jolla, CA, USA) was used for statistical analysis and graphs’ construction. Statistical differences were performed by independent *t*-test at *p* < 0.05.

## 5. Conclusions

Overall, this study upgraded knowledge of genetic polymorphism in sesame through whole-genome resequencing of 410 worldwide accessions. Population structure and genetic diversity analyses classified sesame population into different geographic patterns, consistent with reported potential domestication of the crop from the southern to the middle and northern regions. The key selected genes in MC and NC subgroups were identified for exploitation in sesame improvement. Genotype and environmental conditions have significant impacts on lignan variation in sesame. We identified key loci and 11 candidate genes governing the major sesame lignans (sesamin and sesamolin) variation. Of them, *SiNST1* is the major underlying gene via a non-synonymous SNP (C/A). The “C” allele may favorize lignan and other quality traits accumulation, while the “A” may promote seed coat thickness. Functional characterization in sesame hairy roots revealed that *SiNST1* might function in synergetic action with *SiMYB58*, *SiMYB209*, *SiMYB134*, and *SiMYB276* to control monolignol biosynthetic gene expression at transcriptional levels. Our results may considerably contribute to sesame quality improvement via genomic-assisted breeding.

## Data Availability

The raw resequencing data reported in this paper have been deposited at the Genome Sequence Archive [87], National Genomics Data Center [88], China National Center for Bioinformation/Beijing Institute of Genomics, Chinese Academy of Sciences (GSA: CRA006452), publicly accessible at https://ngdc.cncb.ac.cn/gsa, accessed on 22 November 2021. The RNA-seq data is available at the National Center for Biotechnology Information (NCBI), Sequence Read Archive (SRA), accession PRJNA806786. Other datasets used and/or analyzed during the current study are available from the corresponding author on reasonable request.

## Supplementary Materials

The following supporting information can be downloaded at: https://www.mdpi.com/article/10.3390/ijms24021055/s1.
