# Supplementary material for: Resequencing of 410 Sesame Accessions Identifies SINST1 as the Major Underlying Gene for Lignans Variation
Source: Int J Mol Sci. 2023 Jan 5;24(2):1055. doi: 10.3390/ijms24021055 (PMC9860558; doi:10.3390/ijms24021055)
Supplement: Supplementary file 1 [file ijms-24-01055-s001.zip › Supplementary Figures.pdf]

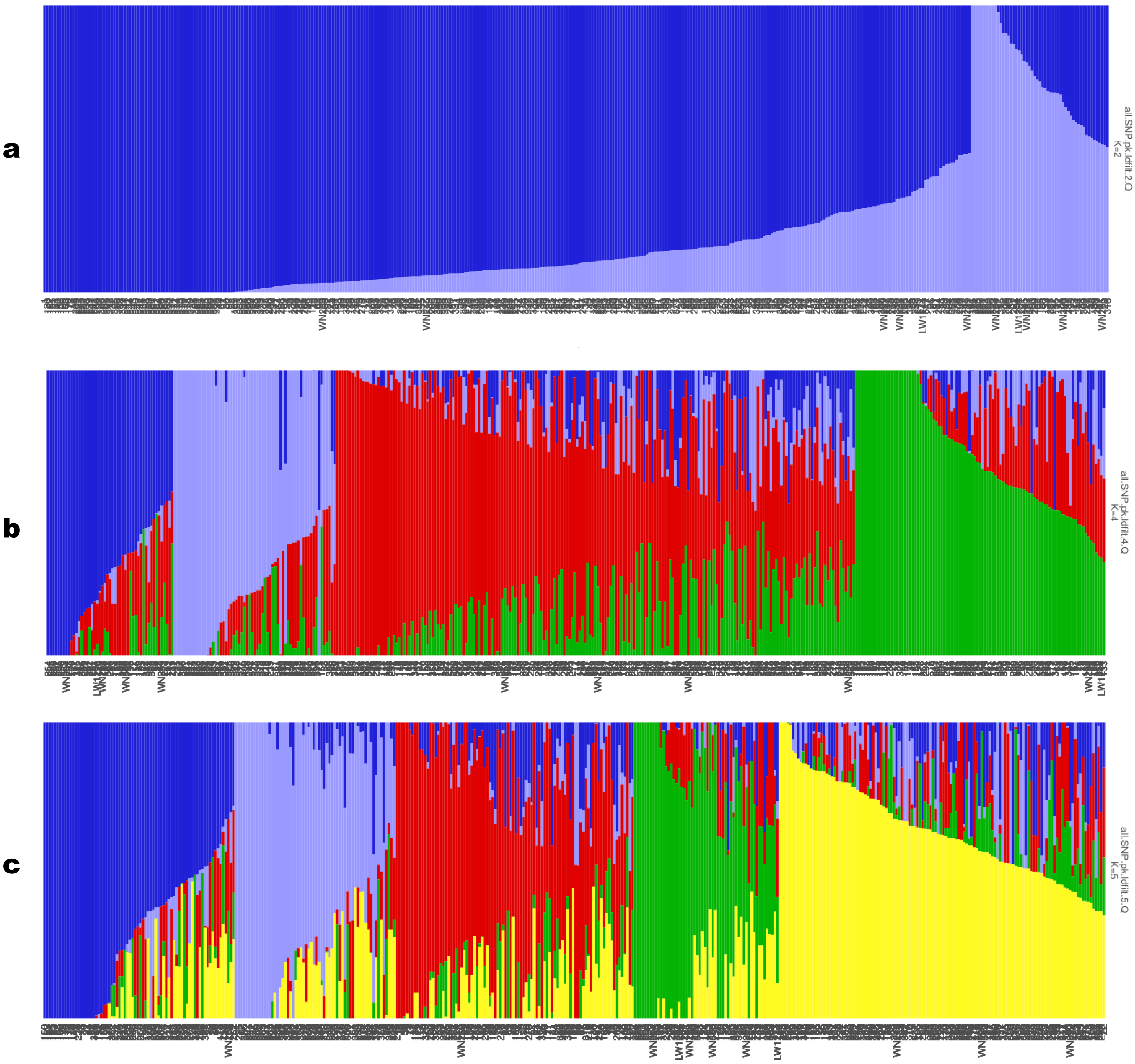

Figure S1. Population structure of 410 sesame accessions. (a) – (C) K = 2, 4, and 5, respectively. The y-axis quantifies subgroups membership and the x-axis lists the different accessions. Each color represents one ancestral population. Each accession is represented by a bar, and the length of each colored segment in the bar represents the proportion contributed by that ancestral population.

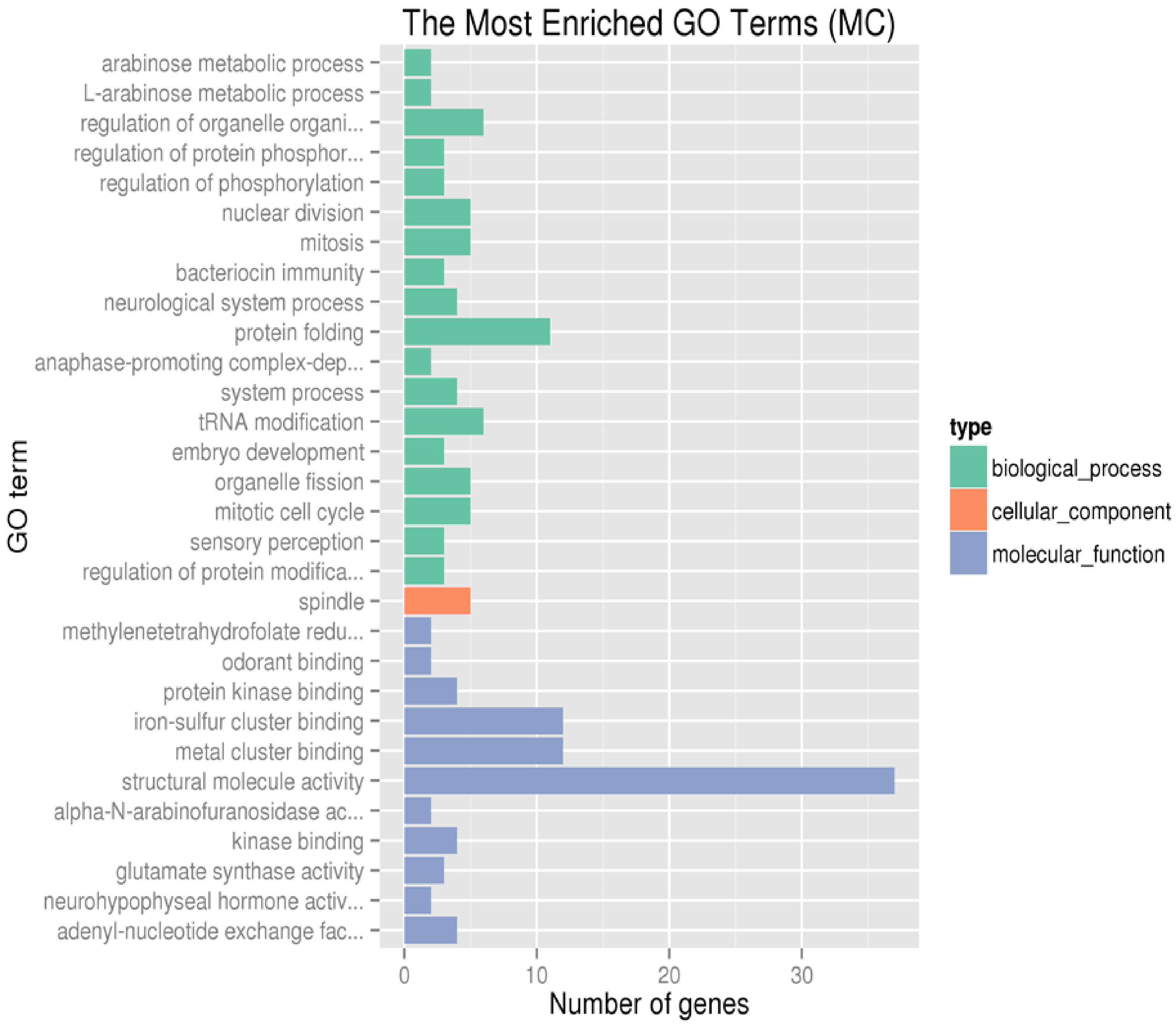

Figure S2. GO (Gene Ontology) analysis results of positively selected genes in MC subpopulation.

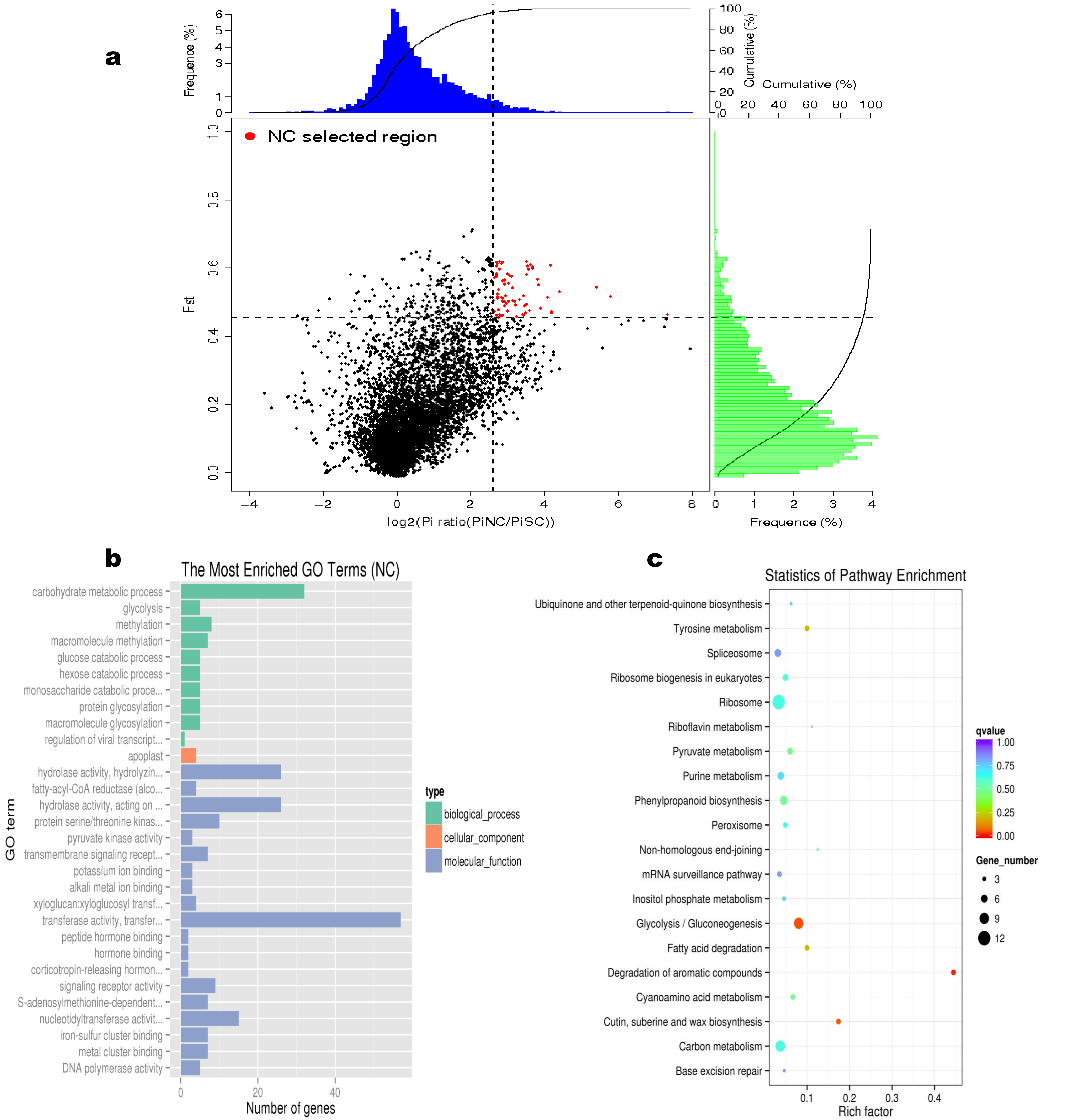

Figure S3. Selective sweeps and functional characterization of positively selected genes in NC subpopulation. (a) Selection signatures in NC. Each red dot indicates a positive selective genomic regions. (b) GO enrichment analysis of selected genes. (c) KEGG analysis and enrichment results. The color ruler and p-values are shown on the

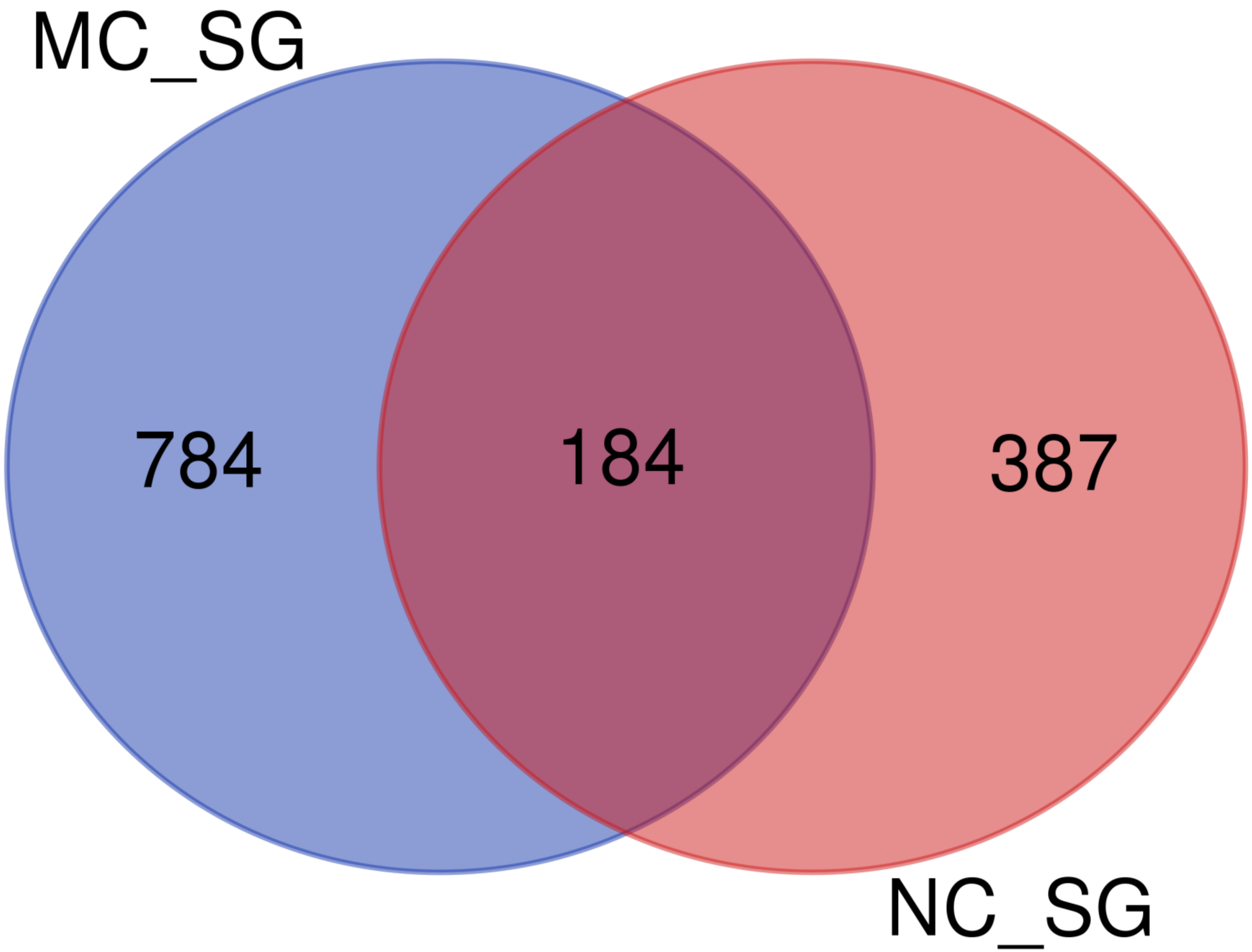

Figure S4. Venn diagram showing the common genes that were positively selected in MC and NC subpopulations.

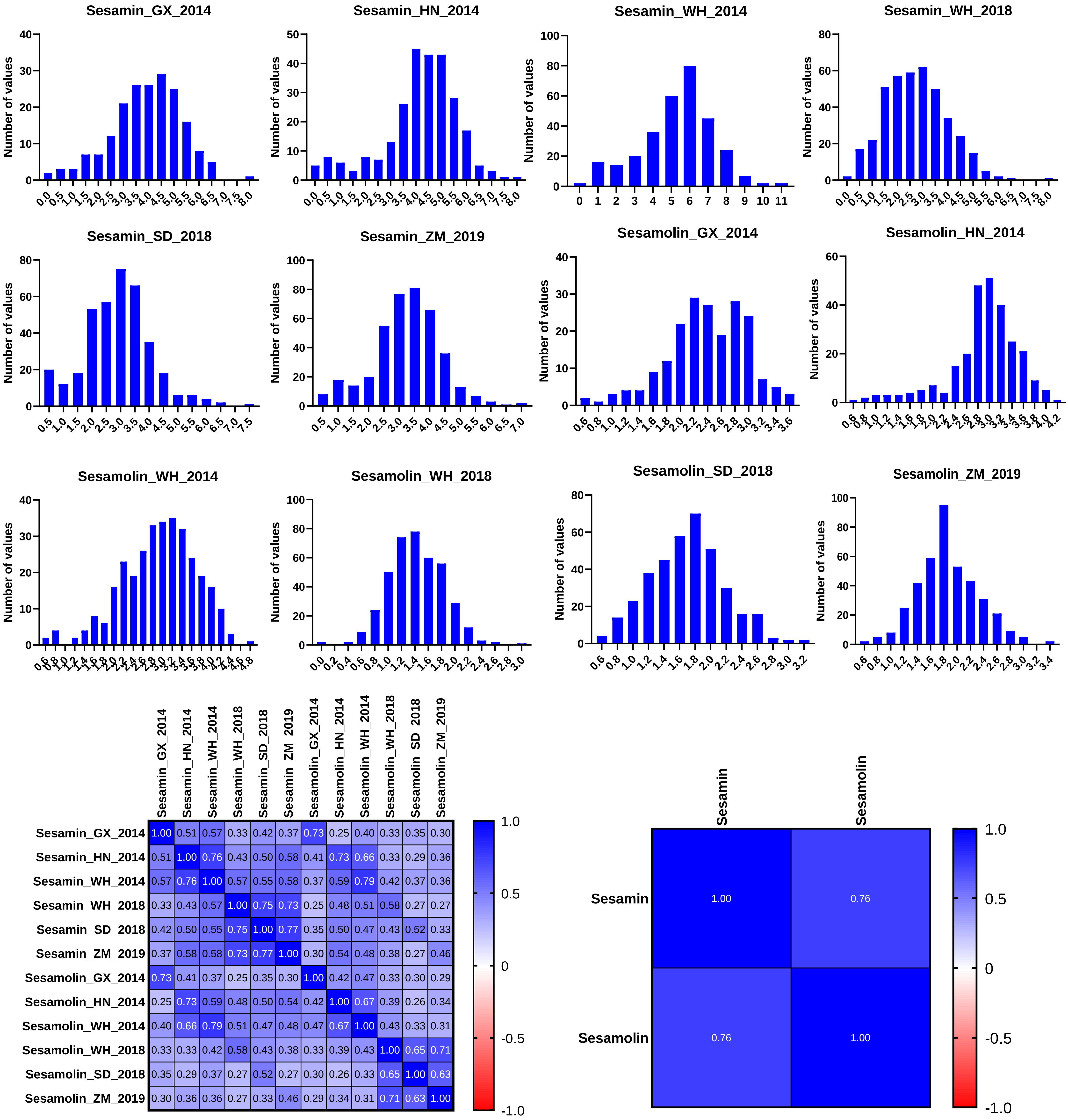

Figure S5. Frequency distribution and correlation between sesamin and sesamolin in six different environments

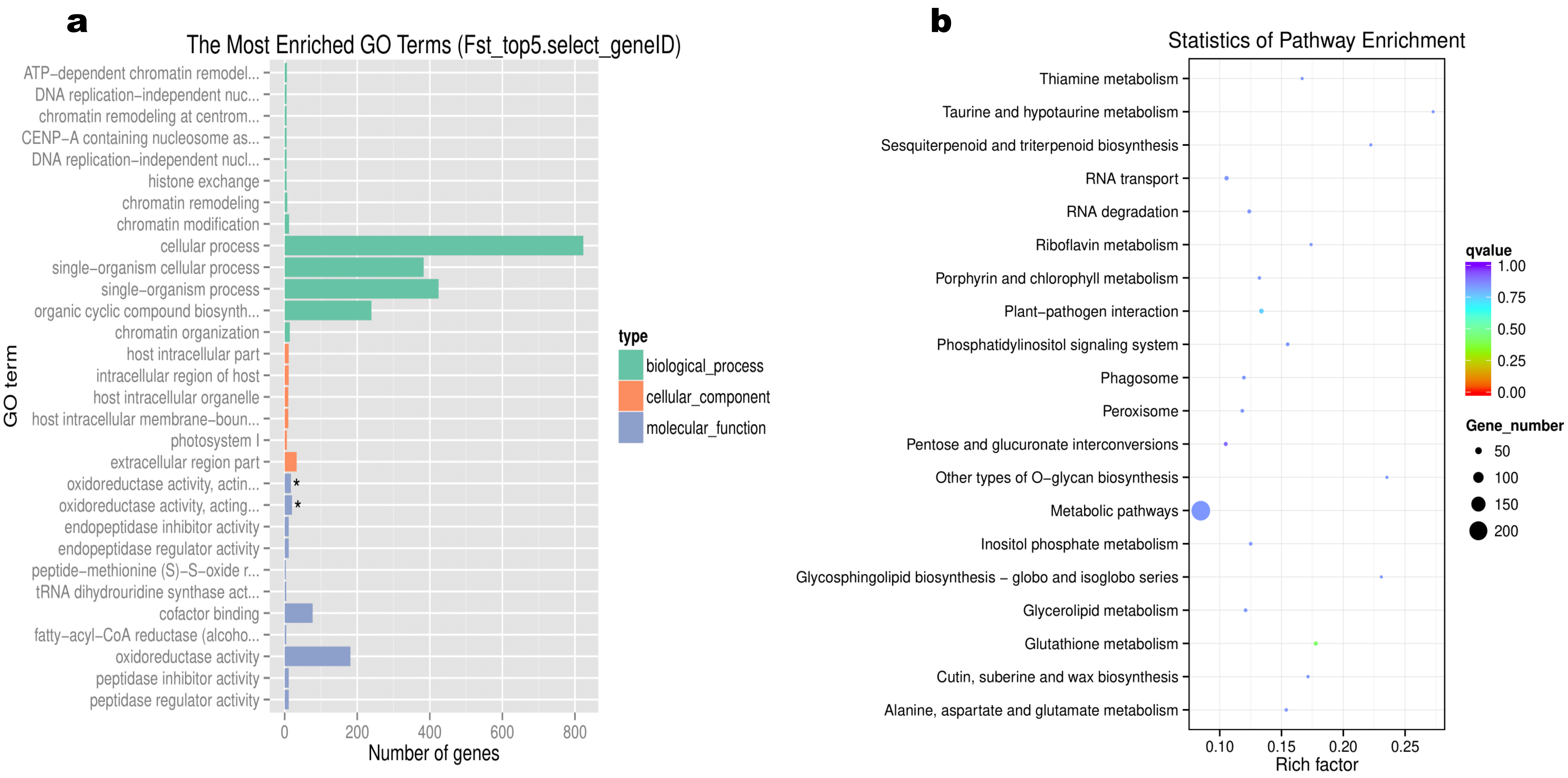

Figure S6. GO terms enrichment (a) and KEGG enrichments results (b) of genes identified at the significant divergent genomic regions between in high-sesamin and low-sesamin content sesame accessions.

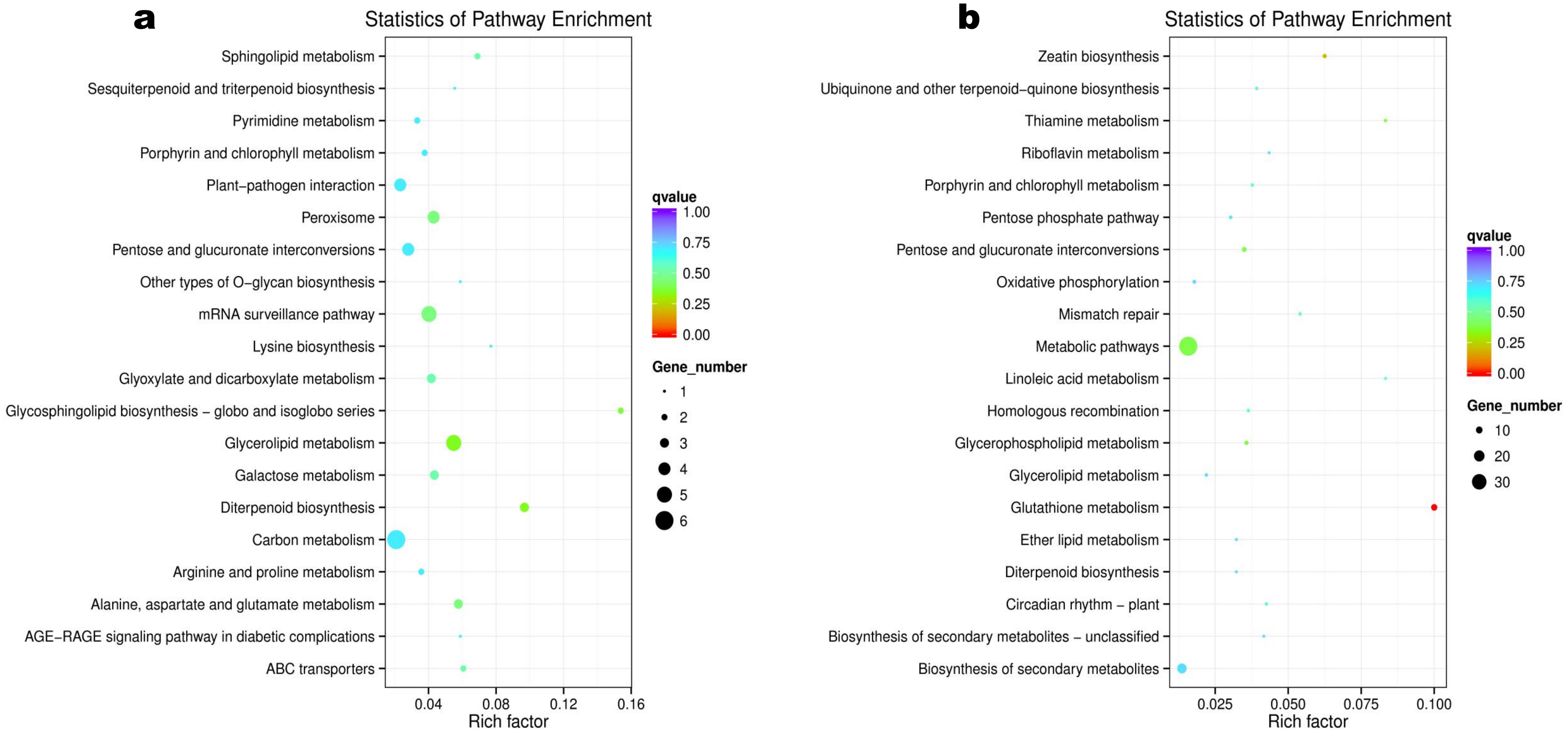

Figure S7. KEGG enrichments results of the candidate selected genes in: (a) high-sesamin content sesame accessions and (b)) low-sesamin content sesame accessions.

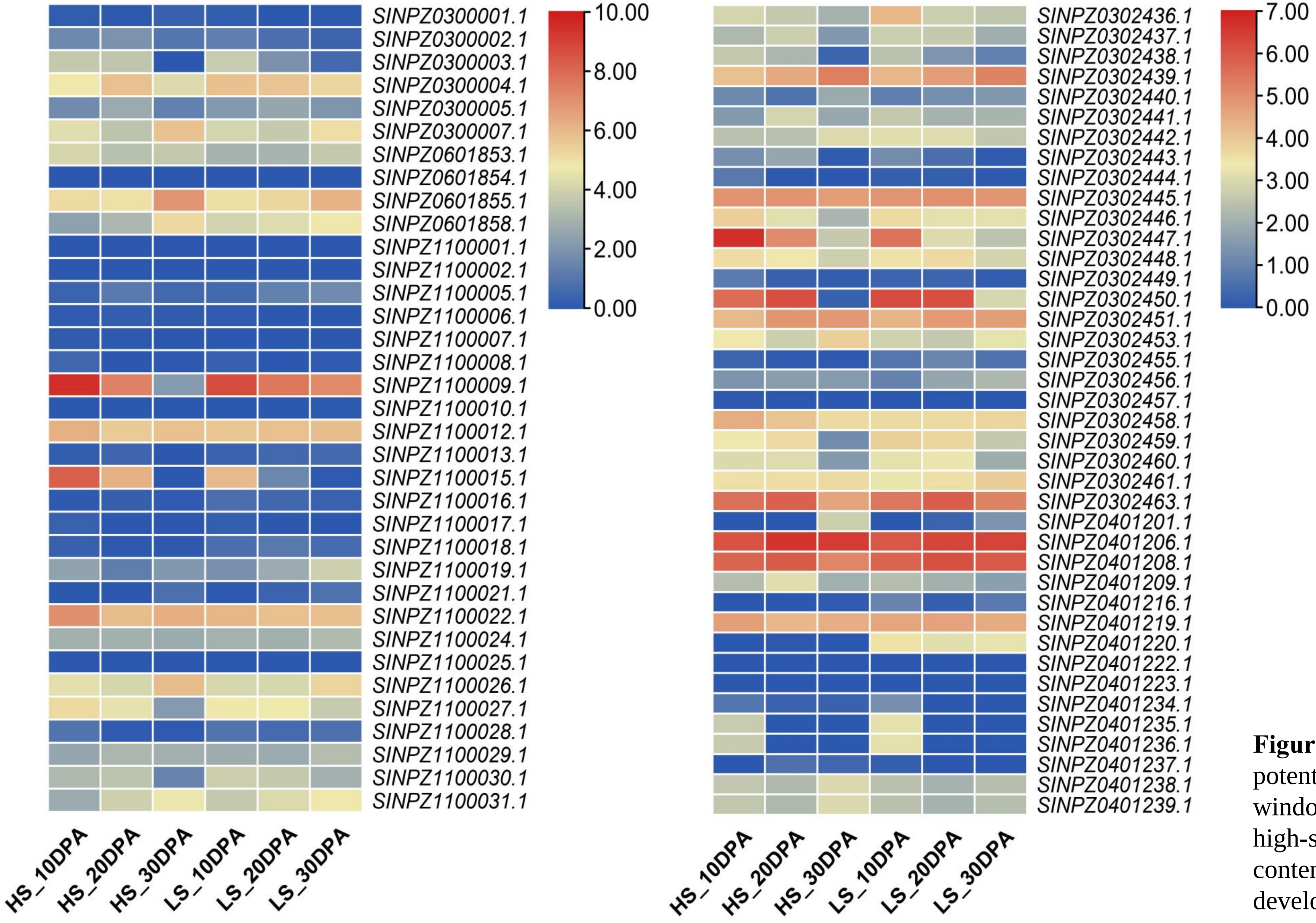

Figure S8. Relative expression of the potential candidate genes detected in the LD windows of non-synonymous SNP loci in a high-sesamin (HS) and low-sesamin (LS) content sesame accession at different seed developmental stages.

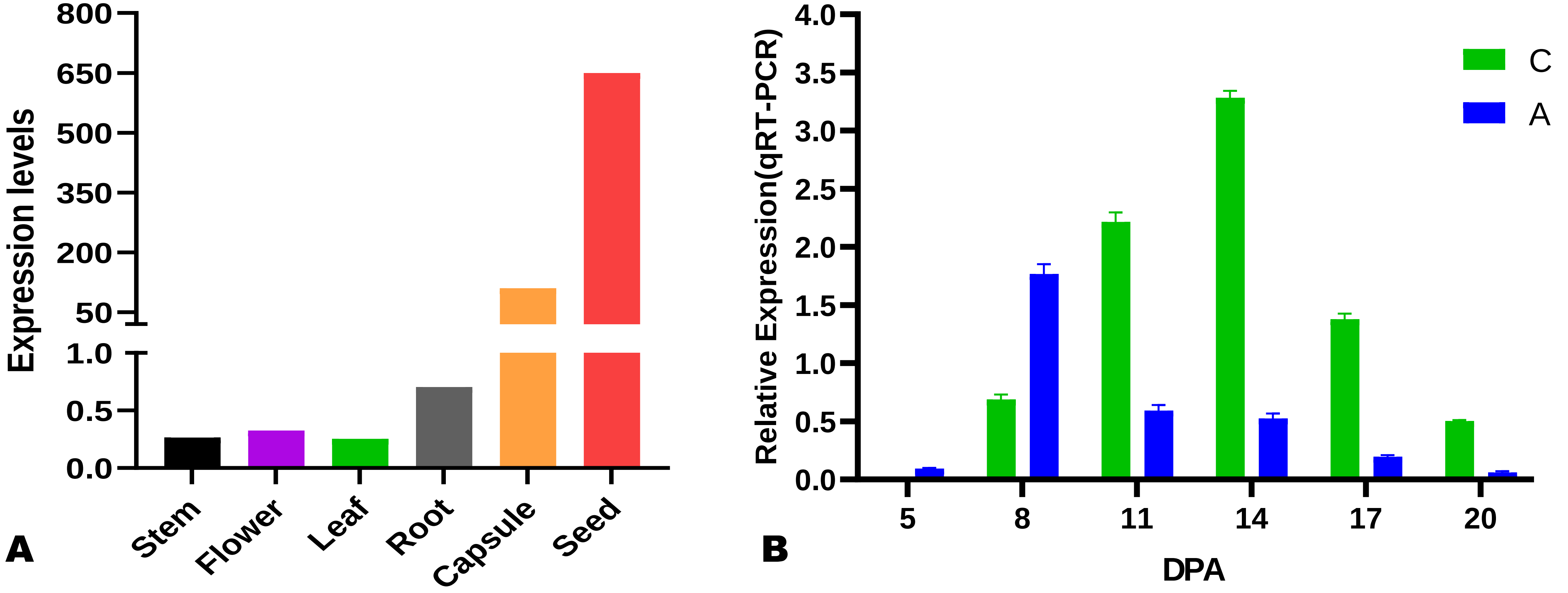

Figure S9. Expression level of *SiNST1* in different organs of sesame (A). Relative expression of *SiNST1* in sesame accessions with “A” and “C” alleles, respectively, at different seed developmental stages (B).

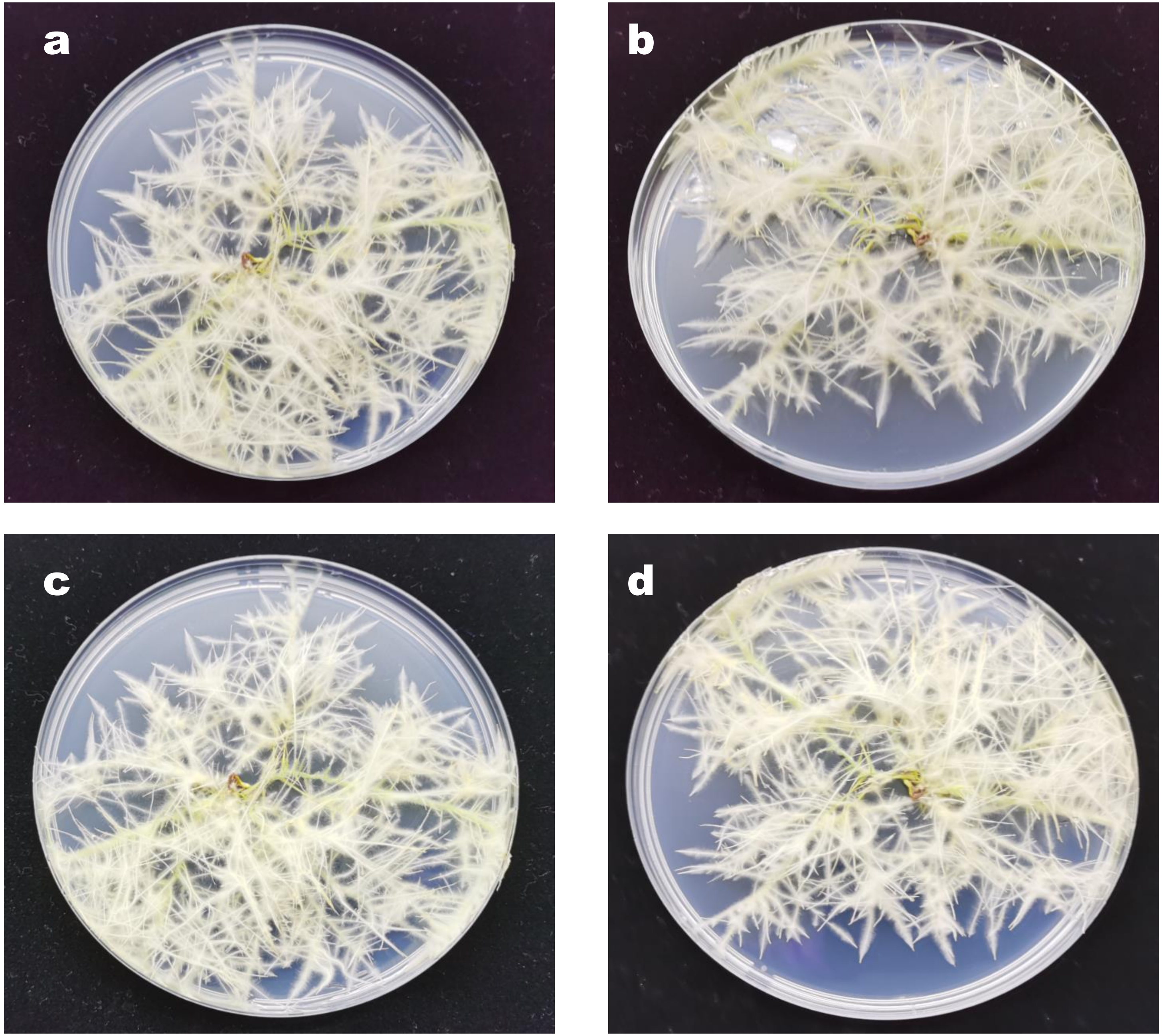

Figure S10: Representative photos of sesame hairy roots. (a) and (b), Control; (c) and (d) SiNST1-overexpessing lines (OE).
